# Mesenchymal stem cells protect against TBI-induced pyroptosis in vivo and in vitro through TSG-6

**DOI:** 10.1186/s12964-022-00931-2

**Published:** 2022-08-18

**Authors:** Zhiming Feng, Shiting Hua, Wangan Li, Jianbang Han, Feng Li, Haijia Chen, Zhongfei Zhang, Yu Xie, Qian Ouyang, Xiaoxiong Zou, Zhizheng Liu, Cong Li, Sixian Huang, Zelin Lai, Xiaolin Cai, Yingqian Cai, Yuxi Zou, Yanping Tang, Xiaodan Jiang

**Affiliations:** 1 Neurosurgery Center, The National Key Clinical Specialty, The Engineering Technology Research Center of Education Ministry of China On Diagnosis and Treatment of Cerebrovascular Disease, Guangdong Provincial Key Laboratory On Brain Function Repair and Regeneration, The Neurosurgery Institute of Guangdong Province, Guangdong-Hong Kong-Macao Greater Bay Area Center for Brain Science and Brain-Inspired Intelligence, Zhujiang Hospital, Southern Medical University, Guangzhou, 510282 China; 2 Emergency Trauma Center, Huizhou First Hospital, Huizhou, China; 3 Guangzhou Saliai Stem Cell Science and Technology Co. Ltd, Guangzhou, China

**Keywords:** TBI, Neuroinflammation, Pyroptosis, hUMSCs, TSG-6

## Abstract

**Background:**

Pyroptosis, especially microglial pyroptosis, may play an important role in central nervous system pathologies, including traumatic brain injury (TBI). Transplantation of mesenchymal stem cells (MSCs), such as human umbilical cord MSCs (hUMSCs), has been a focus of brain injury treatment. Recently, MSCs have been found to play a role in many diseases by regulating the pyroptosis pathway. However, the effect of MSC transplantation on pyroptosis following TBI remains unknown. Tumor necrosis factor α stimulated gene 6/protein (TSG-6), a potent anti-inflammatory factor expressed in many cell types including MSCs, plays an anti-inflammatory role in many diseases; however, the effect of TSG-6 secreted by MSCs on pyroptosis remains unclear.

**Methods:**

Mice were subjected to controlled cortical impact injury in vivo. To assess the time course of pyroptosis after TBI, brains of TBI mice were collected at different time points. To study the effect of TSG-6 secreted by hUMSCs in regulating pyroptosis, normal hUMSCs, sh-TSG-6 hUMSCs, or different concentrations of rmTSG-6 were injected intracerebroventricularly into mice 4 h after TBI. Neurological deficits, double immunofluorescence staining, presence of inflammatory factors, cell apoptosis, and pyroptosis were assessed. In vitro, we investigated the anti-pyroptosis effects of hUMSCs and TSG-6 in a lipopolysaccharide/ATP-induced BV2 microglial pyroptosis model.

**Results:**

In TBI mice, the co-localization of Iba-1 (marking microglia/macrophages) with NLRP3/Caspase-1 p20/GSDMD was distinctly observed at 48 h. In vivo, hUMSC transplantation or treatment with rmTSG-6 in TBI mice significantly improved neurological deficits, reduced inflammatory cytokine expression, and inhibited both NLRP3/Caspase-1 p20/GSDMD expression and microglial pyroptosis in the cerebral cortices of TBI mice. However, the therapeutic effect of hUMSCs on TBI mice was reduced by the inhibition of TSG-6 expression in hUMSCs. In vitro, lipopolysaccharide/ATP-induced BV2 microglial pyroptosis was inhibited by co-culture with hUMSCs or with rmTSG-6. However, the inhibitory effect of hUMSCs on BV2 microglial pyroptosis was significantly reduced by TSG-6-shRNA transfection.

**Conclusion:**

In TBI mice, microglial pyroptosis was observed. Both in vivo and in vitro, hUMSCs inhibited pyroptosis, particularly microglial pyroptosis, by regulating the NLRP3/Caspase-1/GSDMD signaling pathway via TSG-6.

**Video Abstract**

**Supplementary Information:**

The online version contains supplementary material available at 10.1186/s12964-022-00931-2.

## Background

Traumatic brain injury (TBI) is a leading cause of mortality and morbidity worldwide [1]. This devastating injury not only impairs physical and psychological health, but also places a significant financial burden on families and society. TBI pathophysiology is complex and involves major mechanical tissue destruction and multiple secondary injury cascades (e.g., mitochondrial dysfunction, oxidative stress, excitotoxicity, and neuroinflammation) [2]. Clinically, the treatment of TBI mainly includes decompressive craniectomy [3–5], therapeutic hypothermia [6–8], and supportive medical care [9]. However, patient recovery remains limited. Although many preclinical studies have shown that the use of drugs with known anti-inflammatory properties, such as glucocorticoids, progesterone, and erythropoietin immediately following a TBI can be effective in improving outcomes [10, 11], clinical trials have documented the poor efficacy of these anti-inflammatory drugs [12–14]. Thus, new concepts and therapeutic targets are warranted for TBI treatment.

Pyroptosis is a type of programmed cell death associated with an inflammatory response [15]. Numerous studies have demonstrated that pyroptosis is extensively involved in CNS disorders, and the existence of pyroptosis has been confirmed in many cell types in the CNS, including neurons, astrocytes, microglia, and endothelial cells [16]. Microglia are vital mediators of innate immune responses following CNS injury [17]. Pyroptosis of microglia plays a key role in the pathogenesis of multiple CNS diseases, including TBI [18–22]. Drug or gene intervention targeting key molecules of the pyroptosis signaling pathway [such as Nod-like receptor protein 3 (NLRP3), Caspase-1, and Gasdermin D (GSDMD)] can effectively treat TBI and its sequelae [23]. These results suggest that the regulation of pyroptosis following a TBI, especially microglial pyroptosis, may represent a new potential therapeutic strategy.

Transplantation of mesenchymal stem cells (MSCs), especially human umbilical cord MSCs (hUMSCs), is a promising treatment strategy for TBI [24]. Several clinical studies have demonstrated that MSCs have a certain therapeutic effect on neurological function recovery in TBI patients, and no serious adverse effects have been identified [24–26]. Existing studies have revealed that the possible mechanisms of MSC transplantation in the treatment of TBI include immune inflammatory regulation, secretion of nutritional factors or growth factors, promotion of nerve regeneration and angiogenesis, and possible structural and functional integration [27, 28]. In recent years, studies have suggested that MSCs may also play a role in various diseases by regulating cell pyroptosis [29–32]. However, the effect of MSC transplantation on the pyroptosis of brain cells following TBI remains unknown.

Tumor necrosis factor α-stimulated gene-6 (TSG-6) produces a relatively small, secreted protein with a molecular weight of only 35–38 kDa, which primarily consists of two domains [33]. Since the discovery that TSG-6 was secreted by MSCs in 2009 [34], a large number of studies have revealed that TSG-6 mediates immune regulation and repair of tissues [35]. Studies have demonstrated that TSG-6 inhibits neuroinflammation, thus promoting nerve damage repair [36–40]. Our previous study also revealed that in the TBI model, MSCs can promote the repair of CNS injury by increasing the expression of TSG-6 and inhibiting the aggravation of inflammation [41]. MSCs inhibit the inflammatory response of BV2 microglia induced by lipopolysaccharide through TSG-6 [42], enhance M2 polarization of microglia, and alleviate neuroinflammation through TSG-6 [43]. These results suggest that TSG-6, as a powerful anti-neuroinflammatory factor secreted by MSCs, may also inhibit pyroptosis, which represents a key part of the neuroinflammatory response. However, whether TSG-6 secreted by MSCs can regulate pyroptosis in brain cells following TBI remains unclear. Therefore, in the present study, we assessed the inhibitory effects of hUMSCs on TBI-induced pyroptosis and investigated whether TSG-6 secreted from hUMSCs exerts its protective effect by suppressing the activation of the NLRP3/Caspase-1/GSDMD signaling pathway and attenuating pyroptosis in microglia.

## Materials and methods

### Human umbilical cord mesenchymal stem cell isolation, culture, and characterization

hUMSCs were isolated and provided by Guangzhou Saliai Stem Cell Science and Technology Co. Ltd. Thereafter, hUMSCs were cultured in OriCell®Basal Medium (Cyagen, China) supplemented with 10% fetal bovine serum (FBS) (Cyagen). hUMSCs were assessed using flow cytometry to detect cells expressing the typical markers CD73, CD105, and CD90, and cells that were negative for CD45, HLA-DR, CD34, CD11b, and CD9 (Additional file 2: Fig. S1). Third to fifth generation cells were used for subsequent experiments.

### Transfection of hUMSCs with shRNA

hUMSCs at passage 3 were used for the transfection. In brief, hUMSCs were seeded in 6-well culture plates at 2 × 10^5^ cells per well and cultured for 24 h. For TSG-6 downregulation, the cells were then infected with lentivirus shRNA against TSG-6 (sh-TSG-6) or its negative control (sh-NC) (Shanghai Genechem Co., Ltd., Shanghai, China) according to the manufacturer’s instructions. For TSG-6 knockdown lentiviral vector, the two shRNA candidate sequences were sequence 1: CACCGCCATTAGCATCAGCGGTAGTCTCGAGACTACCGCTGATGCTAATGG-C and sequence 2: AAAAGCGGTAGTGGGAGTATTATCACTCGAGTGATAAT-

ACTCCCACTACCGC. RNA and protein were extracted from aliquots of the cells, and real-time quantitative polymerase chain reaction (RT-qPCR) and western blotting were conducted to determine the knockdown of TSG-6(Additional file 3: Fig. S2). Finally, sequence 1 was selected to generate the data presented in the following experiments.

### TBI animal models

Male BALB/c mice (24 g body weight) obtained from the central animal facility of Southern Medical University (Guangzhou, China) were used in this study. All experimental protocols were approved by the Animal Welfare of the Southern Medical University Experimental Animal Center. TBI was induced using a controlled cortical impact device (68099II device, RWD, Shenzhen, China). Briefly, the mice were anesthetized with pentobarbital (40 mg/kg) intraperitoneally and fixed in a stereotaxic frame. Body temperature was maintained at 37 ± 0.5 °C by a heating pad (RWD, Shenzhen, China) during the surgical procedure. A 3.5-mm craniotomy was performed over the right parietal cortex (2.0 mm posterior to anterior and posterior iliac crest, 2.0 mm lateral sagittal suture) with the dura intact. A moderate TBI with a diameter of 3 mm was produced on the head with an impact speed of 4 m/s for 500 ms, retaining the inhibitory effect of 2.0 mm. Mice in the sham group underwent the same craniotomy procedure without any cortical impact. All operations were performed using rigorous aseptic techniques.

### hUMSCs or TSG-6 treatment

BALB/c mice were randomly divided into seven groups: (1) sham, (2) TBI + PBS, (3) TBI + hUMSCs, (4) TBI + sh-TSG-6 hUMSCs, (5) TBI + sh-NC hUMSCs, (6) TBI + 0.5 µg recombinant mouse (rm)TSG-6 (R&D Systems, Oxford, UK), and (7) TBI + 1 µg rmTSG-6. The mice from the sham group were subjected to craniotomy without cortical impact. The remaining mice underwent TBI surgery and were intracerebroventricularly injected with 10 μL of PBS (TBI + PBS group) or 2.5 × 10^5^ hUMSCs (TBI + hUMSCs group), sh-TSG-6 hUMSCs (TBI + sh-TSG-6 hUMSCs group), sh-NC hUMSCs (TBI + sh-NC hUMSCs group), in 10 μL of PBS at 4 h after TBI surgery (Our previous study indicated that MSCs administration at the onset of the inflammatory response after TBI is more likely to be effective [41]). For the TBI + 0.5 µg rmTSG-6 and TBI + 1 µg rmTSG-6 groups, the mice underwent TBI surgery and were intracerebroventricularly injected with 10 μL of rmTSG-6 0.5 and 1 μg, respectively, at 4 h after TBI surgery.

### Behavioral tests

The murine modified neurological severity score (mNSS) was assessed at 12, 24, 48, and 72 h after TBI to assess the clinical severity of the disease [44]. Briefly, mice were subjected to a four-part test probing motor, sensory, reflex, and balance deficits. An overall composite score was then assigned to score the impairment, with a normal to maximal deficit score of 0–18.

### Assessment of cerebral edema

Brain water content (BWC) was assessed at 24 and 48 h following TBI surgical treatment. After anesthesia and sacrifice, the brains were collected instantly and cut into two hemispheres following the midline, and the cerebella were removed. Ipsilateral hemispheres were kept in a pre-weighed piece of aluminum foil to determine the wet weight, and then dried at 100 °C for 24 h in an electric oven and re-weighed on the same piece of foil. The brain water percentage was calculated as follows: (wet weight − dry weight)/(wet weight).

### Terminal deoxynucleotidyl transferase-mediated dUTP-biotin nick end labeling (TUNEL) staining

The cerebral cortex was harvested and sliced, and TUNEL staining was performed to identify apoptosis (One Step TUNEL Apoptosis Assay Kit, Beyotime, China). In brief, slices were incubated with fluorescein-dUTP for 1 h at 37 °C to identify apoptotic cell nuclei and 4′,6-diamidino-2-phenylindole (DAPI) for 5 min at 37 °C to stain all cell nuclei. The apoptosis index (AI), which is the number of TUNEL-positive cells divided by the total number of cells per field, was calculated. Each AI was evaluated in a total of 15 randomly selected fields.

### Double immunofluorescence staining

TBI mice were anesthetized before receiving 30 mL cold PBS and 30 mL PBS containing 4% paraformaldehyde through transcardial infusion. Thereafter, the brains were collected, post-fixed, and paraffin-embedded, and consecutive coronal sections were cut at 2.5 mm intervals from − 1.0 to − 3.5 mm of the bregma to retrieve the entire lesioned cortex. The brain slices were deparaffinized and microwave-boiled in 10 mM citrate buffer (pH 6.0) to expose the antigens before being blocked with 10% normal goat serum. Primary antibodies, Mouse anti-Iba-1 (GB12105, Servicebio, 1:100), rabbit anti-NLRP3 (GB114320, Servicebio, 1:100), rabbit anti-Caspase-1 p20 (AF4005, Affinity, 1:100), and rabbit anti-GSDMD (ab219800, Abcam, 1:100) were added to the sections and incubated overnight at 4 °C. After three 5-min rinses with PBS, the sections were incubated with FITC-conjugated goat anti-mouse IgG&L (ab6785, Abcam, 1:1000) or Cy5-conjugated goat anti-rabbit IgG&L (ab6564, Abcam, 1:1000) at 37 °C for 1 h before being mounted with Fluoroshield containing DAPI. Images of the brain sections were obtained by confocal microscopy. In a blinded manner, the number of positive cells around the damaged areas was counted (8–10 slices each brain, 500 μm apart).

### Activation of hUMSCs using TNF-α

Tumor necrosis factor alpha (TNF-α) was utilized to stimulate hUMSCs before they were employed in co-cultures. In brief, 2 × 10^5^ hUMSCs were seeded per well in 6-well plates with 2 mL of DMEM/F12 supplemented with 10% FBS and cultured for 1 day. Thereafter the medium was changed to DMEM/F12 with 2% FBS and 10 ng/mL TNF-α (R&D Systems, USA). After an 18-h incubation, the cells were trypsinized for 3 min at 37 °C with 0.25% trypsin (Gibco). RNA and protein were extracted from cell aliquots, and RT-qPCR and western blotting were performed to confirm the increased expression of TSG-6 (Additional file 4: Fig. S3).

### BV2 cell culture

The BV2 murine microglial cell line was cultured in DMEM supplemented with 10% heat-inactivated FBS, 100 μg/mL streptomycin, and 100 U/mL penicillin (HyClone). A 6-well transwell system (0.4 μm pore size membrane; Corning) was utilized to evaluate the effects of hUMSCs and TSG-6 on lipopolysaccharide (LPS)/ATP-stimulated BV2 microglial cells. A total of 5 × 10^5^ BV2 cells were seeded in the lower chamber with or without 1 μg/mL LPS and 5 mM ATP for 12 h, with one of the following treatments in the upper chamber: (1) control medium (LPS + ATP group); (2) 1 × 10^5^ activated hUMSCs (LPS + ATP + hUMSCs group); (3) 1 × 10^5^ activated hUMSCs transfected with sh-TSG-6 lentivirus (LPS + ATP + sh-hUMSCs group); (4) 1 × 10^5^ activated hUMSCs transfected with negative control lentivirus (LPS + ATP + sh-NC group); (5) 50 ng/mL rmTSG-6; (6) 100 ng/mL rmTSG-6; (7) 200 ng/mL rmTSG-6.

### Propidium iodide (PI) staining

BV2 cells were incubated with 1 μg/mL of PI staining reagent (Beyotime Biotechnology Co., Ltd., Shanghai, China) and DAPI. PI-positive cells were photographed after 30 min of incubation at 37 °C, demonstrating red fluorescence. ImageJ software was utilized to count the number of PI-positive cells in a blinded manner and data were presented as a percentage of total cells.

### Caspase-1 activity detection

The Caspase-1 Activity Assay Kit (Solarbio, Beijing) was utilized to measure Caspase-1 activity. The following steps were conducted exactly as recommended by the manufacturer: First, 2–10 × 10^6^ cells were lysed for 10 min on ice in 50–100 μL lysis buffer. The tissues (3–10 mg) were added to 100 μL lysis buffer, homogenized with a tissue homogenizer, and centrifuged. The supernatant was retained. The Bradford method was used to determine protein concentrations, assuring that the protein concentration was 1–3 μg/μL. A *p*-nitroaniline colorimetric reference was used to create a standard curve. The optical density of the specimen was then measured at 405 nm using a microplate reader (Molecular Devices). The percentage of Caspase-1 activity changes was calculated by the ratio of OD405 of the experimental wells to that of the normal wells.

### LDH release detection

Following deep anesthesia, mice were sacrificed for blood serum collection (centrifugation at 3000×*g* for 10 min), and the blood serum lactate dehydrogenase (LDH) release was evaluated. For the BV2 cells, supernatant from serum-free media was filtered through 0.2-μm syringe filters to detect LDH release. A commercially available kit (Solarbio, Beijing) was applied for detection. Blood serum or supernatant (100 μL) was added to 96-well plates, followed by the reaction mixture, then incubated in the dark for 30 min at 37 °C. LDH concentration was quantified by measuring the absorbance at 490 nm.

### Western blotting

Following total protein extraction from BV2 cells and brain tissues, the total protein concentration was detected using a BCA Protein Assay Kit (Thermo). The protein samples (representing a total of 30 mg) were divided by sodium dodecyl sulfate–polyacrylamide gel electrophoresis and then transferred onto a polyvinylidene fluoride membrane (Millipore Corporation, Billerica, MA, USA). The membranes were blocked for 2 h at 37 °C with 5% non-fat milk and 0.05% Tween-20, then incubated overnight at 4 °C with Mouse anti-GAPDH (60004-1-Ig, Proteintech, 1:5000), rabbit anti-NLRP3 (#15101, CST, 1:1000), rabbit anti-pro-Caspase-1 (AF5418, Affinity, 1:1000), rabbit anti-Caspase-1 p20 (AF4005, Affinity, 1:1000), rabbit anti-GSDMD (ab219800, Abcam, 1:1000), and rabbit anti-cleaved-GSDMD (#10137, CST, 1:1000). Subsequently, the membranes were washed three times and incubated for 1 h at 37 °C with horseradish peroxidase-conjugated goat anti-mouse antibody (G1214-100UL, Servicebio, 1:5000) or horseradish peroxidase-conjugated goat anti-rabbit secondary antibodies (G1213-100UL, Servicebio, 1:5000) at a dilution of 1:5000. Finally, the protein bands were developed using an enhanced chemiluminescence detection reagent (Pierce, Rockford, IL, USA), which were quantified using Quantity One 1-D analysis software (Version 4.4, Bio-Rad, Hercules, CA, USA) and normalized to the internal control GADPH. All immunoblots were independently repeated at least three times, and the relative protein expression was represented as a ratio to the internal control.

### RT-qPCR

Total RNA was extracted from hUMSCs using the Total RNA Extraction Kit (Solarbio, Beijing, China). First-strand cDNA was synthesized using random primers through iScript cDNA synthesis kits. Quantitative PCR was carried out using an LC480 PCR instrument (LightCycler®480 II) with the SYBR Green real-time PCR method (TaKaRa) and the manufacturer’s three-stage program settings. Each sample was analyzed in triplicate, and the analysis was performed using the ΔΔCt method. GAPDH was amplified in parallel as an internal control. The primer sequences are as Table 1.

**Table 1:** Primer sequences

Gene	Primer sequence (5′–3′)	
name	Forward primer	Reverse primer
TSG-6	GCTGCTGGATGGATGGCTAA	CCTGGCTTCACAATGGGGTA
GAPDH	AAATCAAGTGGGGCGATGCT	AGCCAAATTCGTTGTCATACTTCT

### Enzyme-linked immunosorbent assay (ELISA)

Total protein concentrations were detected using a BCA Protein Assay Kit (Thermo Fisher Scientific, Carlsbad, CA, USA). The levels of interleukin (IL)-1β, IL-18, and TNF-α were detected using ELISA kits (MULTI SCIENCES-Bio, Hangzhou, China) according to the manufacturer’s instructions.

### Statistical analyses

Data are presented as mean ± standard deviation of five independent experiments. Analyses were performed with SPSS 20 software (IBM, New York, NY, USA) using Student’s t-test or one-way analysis of variance as appropriate. Statistical significance was set at *p* < 0.05.

## Results

### TBI-induced pyroptosis in mice models

**Fig. 1:**
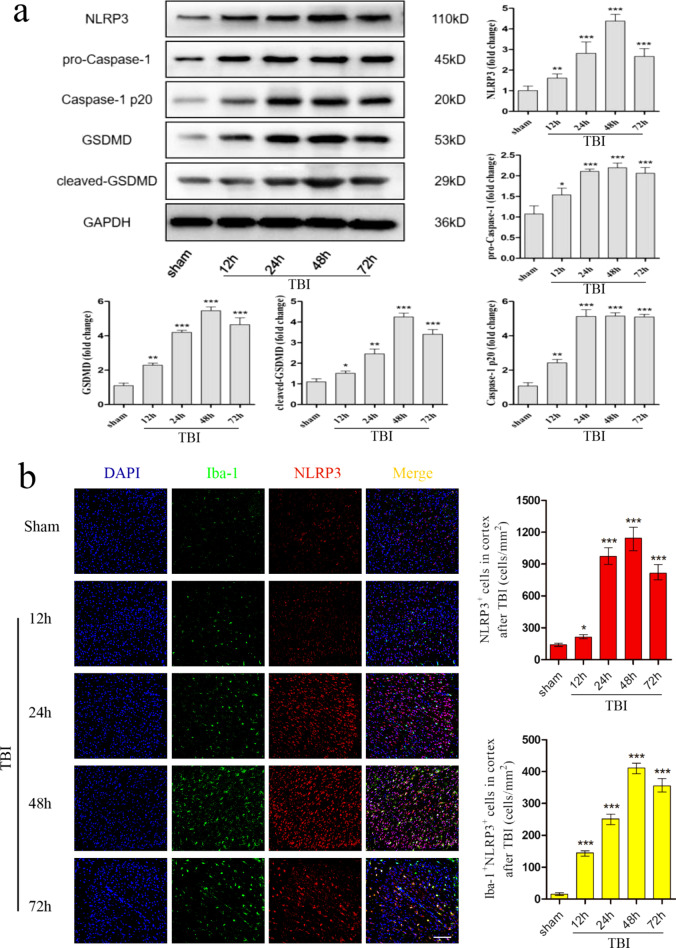

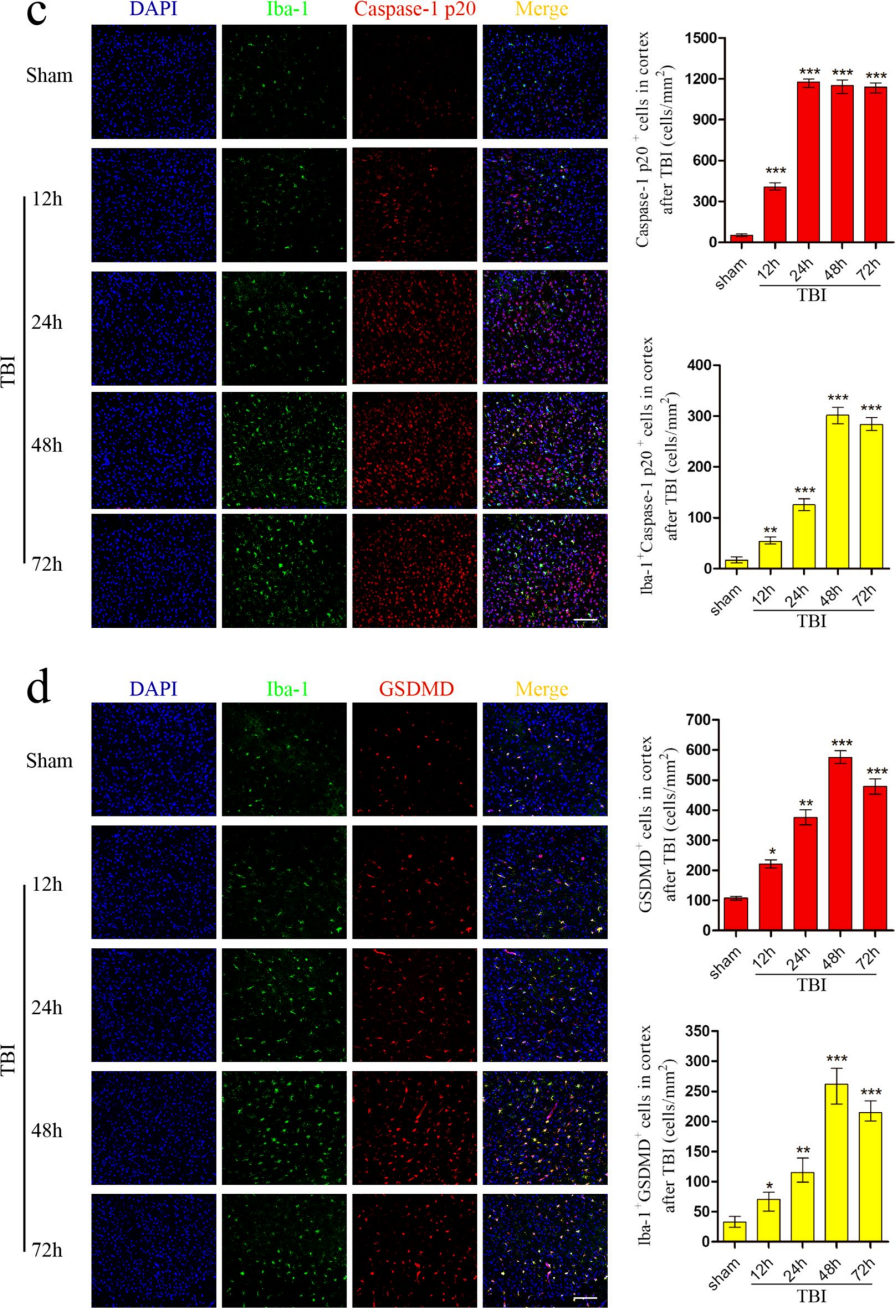
Pyroptosis in the murine TBI model. To assess the expression of pyroptosis-related proteins, TBI mice were sacrificed and brain tissues were removed at 12 h, 24 h, 48 h and 72 h post-TBI surgery. **a** Representative protein bands and corresponding grayscale values of NLRP3, pro-Caspase-1, Caspase-1 p20, GSDMD, and cleaved-GSDMD performed by western blotting. **b**–**d** Representative double immunofluorescence staining photographs and quantification of Iba-1 (green) with NLRP3 (red), Caspase-1 p20 (red), or GSDMD (red) in the injured cortex. Scale bar = 50 μm. All data are represented as means ± SD (n = 5 mice/group) and compared by *t*-test. *p < 0.05, **p < 0.01, ***p < 0.001 versus sham group

To determine whether pyroptosis occurred in the murine TBI model and presented time dependence, inflammation was assessed in the cortex by western blotting and double immunofluorescence staining. As represented in Fig. 1a, the expression of NLRP3, pro-Caspase-1, Caspase-1 p20, GSDMD, and cleaved-GSDMD at 12, 24, 48, and 72 h post-TBI was detected by western blotting. The protein level of NLRP3 gradually increased after TBI, peaking at 48 h. The protein level of both pro-Caspase-1 and Caspase-1 p20 gradually increased after TBI, peaked at 24 h, and then stabilized. The protein level of both GSDMD and cleaved-GSDMD gradually increased after TBI, and peaked at 48 h. Consistently, double immunofluorescence staining revealed similar protein expression intensities of NLRP3, Caspase-1 p20 and GSDMD as western blotting. More importantly, immunofluorescence results also demonstrated that the increased expression of GSDMD coincided with Iba-1 (a marker of microglia) and was distinctly observed at 48 h, which suggested that cell pyroptosis mainly occurred in microglia (Fig. 1b–d).

### TSG-6 secreted by hUMSCs improved neurological recovery and reduced BWC following TBI

**Fig. 2:**
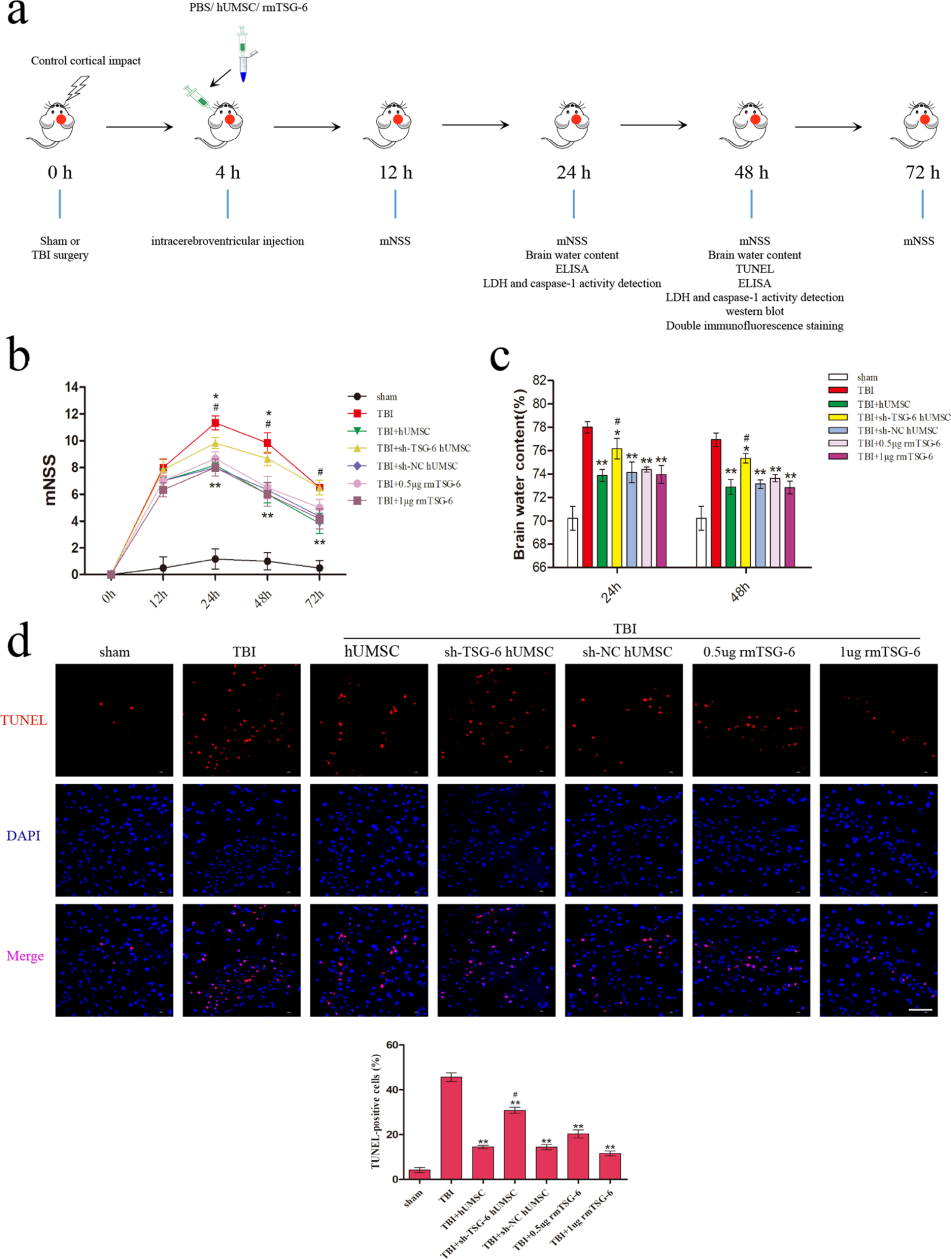
Effect of hUMSCs secreting TSG-6 on neurological recovery and brain water content following TBI. TBI mice were intracerebroventricularly injected with 10 μL of PBS, 0.5 µg rmTSG-6 or 1 µg rmTSG-6, or 2.5 × 10^5^ hUMSCs, sh-TSG-6 hUMSCs, sh-NC hUMSCs, in 10 μL of PBS at 4 h after TBI surgery. **a** Experimental design timeline. **b** Neurological function was assessed by mNSS at 12, 24, 48, and 72 h after TBI. *p < 0.05, sh-TSG-6 hUMSC group versus TBI group; ^#^*p* < 0.05, sh-TSG-6 hUMSC group versus sh-NC hUMSC group; **p < 0.01, hUMSC group, sh-NC hUMSC group, 0.5 µg rmTSG-6 group, or 1 µg rmTSG-6 versus TBI group. **c** Brain water content of ipsilateral hemispheres was also measured at 24 and 48 h after TBI. **d** Representative photographs and quantification of apoptotic cells in the damaged cerebral cortex of mice was measured by TUNEL staining at 48 h post-TBI. Scale bar = 50 μm. All data are represented as the mean ± SD (n = 6 mice/group) and compared by one-way ANOVA, followed by the *Tukey’s *post hoc test. *p < 0.05, **p < 0.01, versus TBI group; ^#^*p* < 0.05, sh-TSG-6 hUMSC group versus sh-NC hUMSCgroup

To assess the effects of TSG-6 secreted by hUMSCs in TBI mice, we first knocked down the expression of TSG-6 in hUMSCs by transfecting shRNA targeting TSG-6 (Additional file 3: Fig. S2). Intracerebroventricular administration of hUMSCs or rmTSG-6 was performed 4 h after TBI. The experimental timeline is shown in Fig. 2a. Next, neurological function was analyzed by mNSS at 12, 24, 48, and 72 h. As shown in Fig. 2b, hUMSC transplantation or treatment with rmTSG-6 significantly reduced mNSS compared to that in the TBI group. Transplantation with sh-TSG-6 hUMSCs significantly increased mNSS compared with that in the sh-NC hUMSC group. Whilst mNSS of TBI mice in the sh-NC hUMSC group and sh-TSG-6 hUMSC group were significantly different from each other, the sh-TSG-6 hUMSC group was still significantly different from the TBI group. The BWC of the ipsilateral hemispheres was also measured at 24 and 48 h after TBI. As represented in Fig. 2c, hUMSC transplantation or treatment with rmTSG-6 significantly reduced BWC, compared to that in the TBI group. And the BWC reduction effects of rmTSG-6 treatments in the TBI mouse was dose-dependent. Transplantation with knocked down TSG-6 hUMSCs significantly increased BWC as compared to that in the sh-NC hUMSC group. Whilst BWC of TBI mice in the sh-NC hUMSC group and sh-TSG-6 hUMSC group were significantly different from each other, the sh-TSG-6 hUMSC group was still significantly different from the TBI group. In addition, cortical damage in mice was measured by TUNEL staining 48 h post-TBI. As represented in Fig. 2d, hUMSC transplantation or treatment with rmTSG-6 significantly reduced cell apoptosis in TBI mice brains. And the anti-apoptosis effects of rmTSG-6 treatments in the TBI mouse was dose-dependent. But transplantation with TSG-6 knockdown hUMSCs significantly increased cell apoptosis in TBI mice brains as compared to that in the sh-NC group. Whilst cell apoptosis of TBI mice brains in the sh-NC hUMSC group and sh-TSG-6 hUMSC group were significantly different from each other, the sh-TSG-6 hUMSC group was still significantly different from the TBI group.

### hUMSCs influenced cytokine levels in injured cortex by secreting TSG-6

**Fig. 3:**
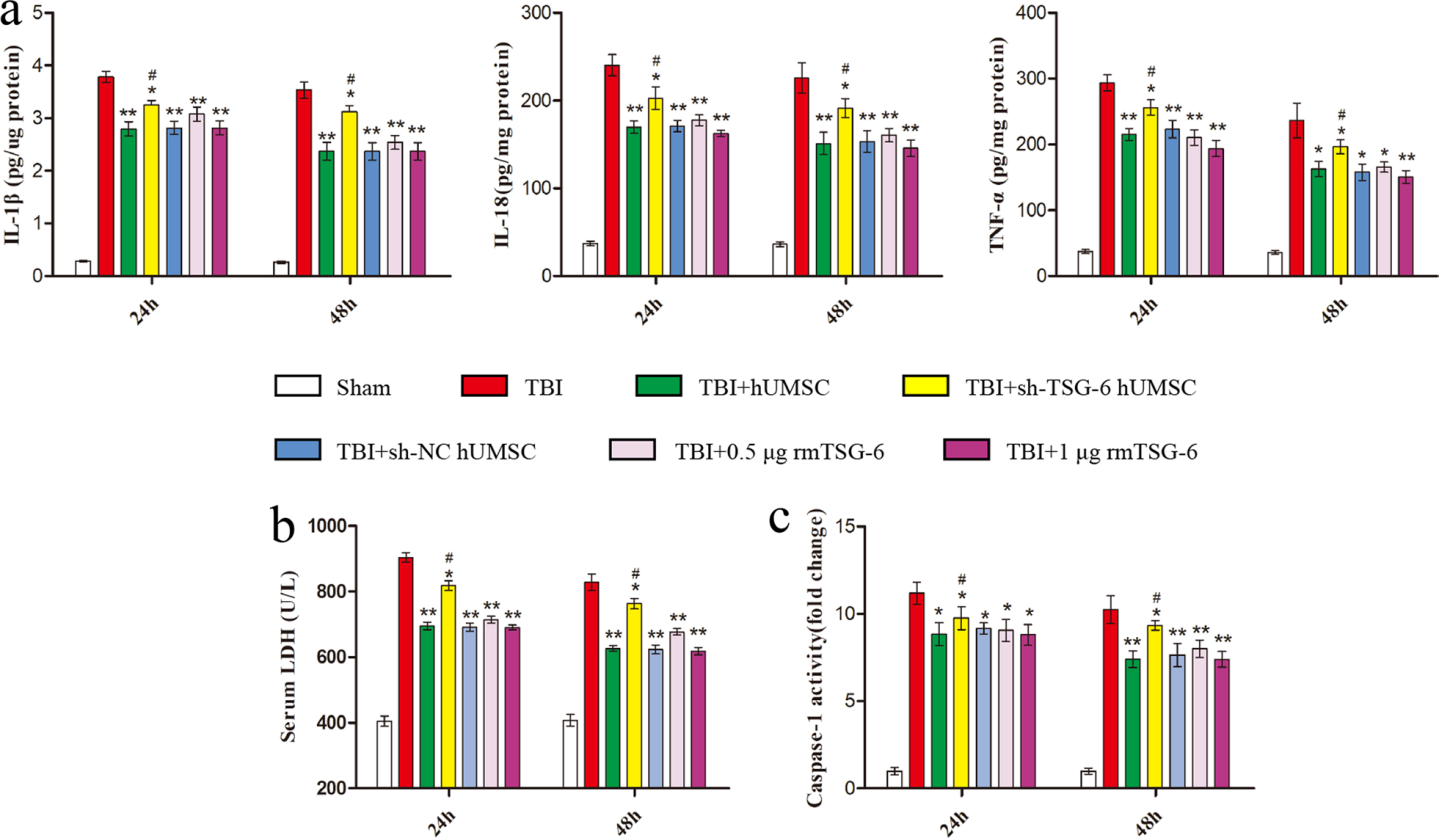
Influence of hUMSCs secreting TSG-6 on inflammatory cytokine expression in the cortex following TBI. TBI mice were intracerebroventricularly injected with 10 μL of PBS, 0.5 µg rmTSG-6 or 1 µg rmTSG-6, or 2.5 × 10^5^ hUMSCs, sh-TSG-6 hUMSCs, sh-NC hUMSCs, in 10 μL of PBS at 4 h after TBI surgery. **a** The protein levels of IL-1β, IL-18, and TNF-α in the cerebral cortex of mice was measured by an ELISA assay at 24 and 48 h post-TBI. Serum LDH (**b**) and Caspase-1 activity (**c**) in brain cortex was also detected 24 h and 48 h post-TBI. All data are represented as means ± SD (n = 6 mice/group) and compared by one-way ANOVA, followed by the *Tukey’s *post hoc test. *p < 0.05, **p < 0.01, versus TBI group; ^#^*p* < 0.05, sh-TSG-6 hUMSC group versus sh-NC hUMSC group

To compare the expression levels of inflammatory factors in the cortex, we measured a series of pro-inflammatory cytokines using an ELISA kit. As represented in Fig. 3a, hUMSC transplantation or treatment with rmTSG-6 significantly reduced the protein levels of IL-1β, IL-18, and TNF-α in the brain cortex of mice at 24 h and 48 h post-TBI. And the anti-inflammatory effects of rmTSG-6 treatments in the TBI mice was dose-dependent. However, the anti-inflammatory effects of hUMSCs were compromised following TSG-6-shRNA treatment. Whilst pro-inflammatory cytokines of TBI mice brains in the sh-NC hUMSC group and sh-TSG-6 hUMSC group were significantly different from each other, the sh-TSG-6 hUMSC group was still significantly different from the TBI group. Serum LDH was measured at 24 and 48 h after TBI to determine cellular LDH leakage caused by brain damage. As represented in Fig. 3b, LDH levels were evidently decreased in the hUMSC and rmTSG-6 groups, indicating the protective role of hUMSCs and TSG-6 during the TBI process. Caspase-1 activity in the brain cortex was also assessed at 24 and 48 h after TBI. As represented in Fig. 3c, the TBI-induced increase in activated Caspase-1 was significantly reduced by hUMSC transplantation and treatment with rmTSG-6. And the reduction effect of rmTSG-6 treatments on Caspase-1 activity in the TBI mice was dose-dependent. However, transplantation with TSG-6 knockdown hUMSCs significantly increased Caspase-1 activity as compared to that in the sh-NC group. Whilst Caspase-1 activity of TBI mice brains in the sh-NC hUMSC group and sh-TSG-6 hUMSC group were significantly different from each other, the sh-TSG-6 hUMSC group was still significantly different from the TBI group.

### hUMSCs suppressed the NLRP3/Caspase-1/GSDMD signaling pathway and reduced microglia pyroptosis in injured cortex by secreting TSG-6

**Fig. 4:**
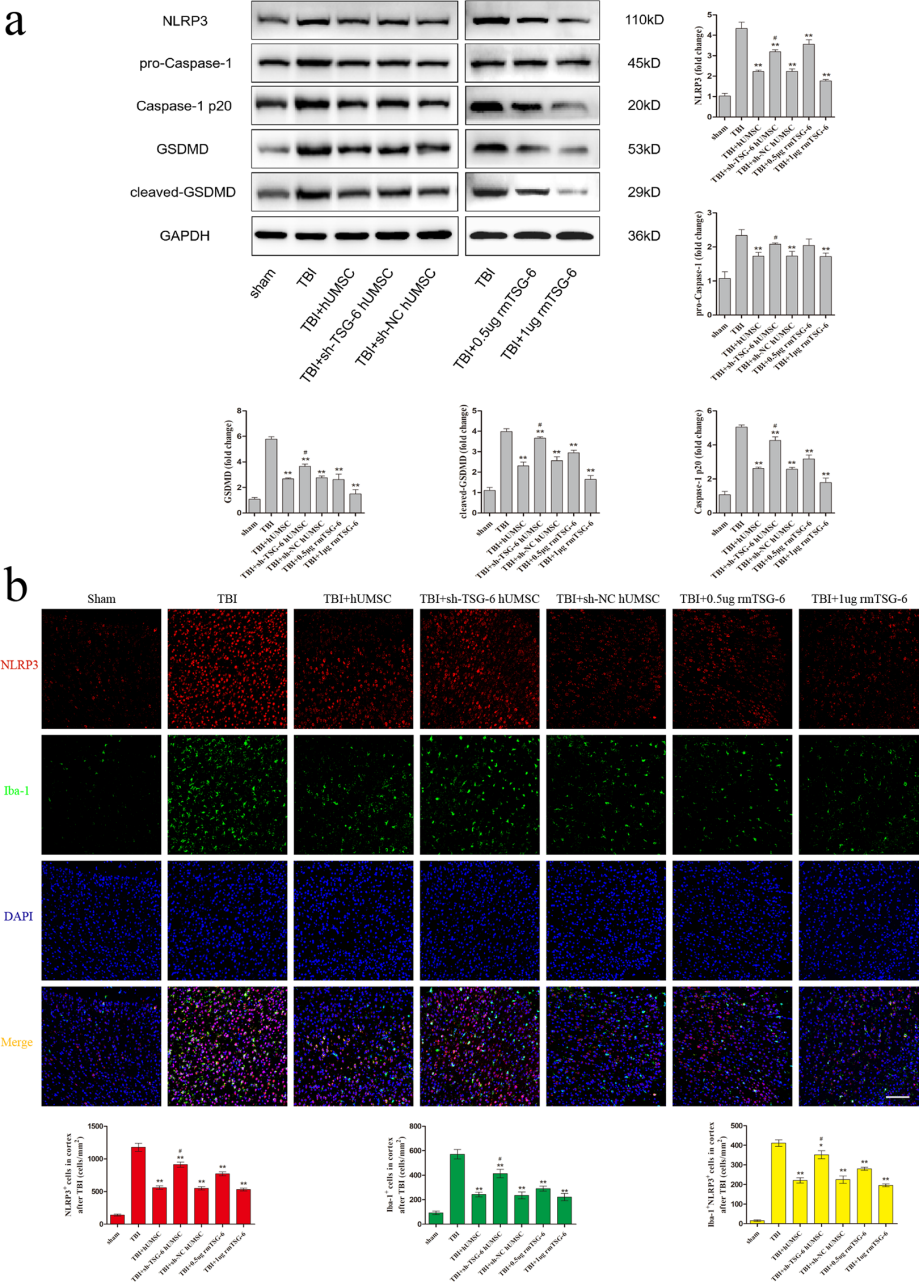

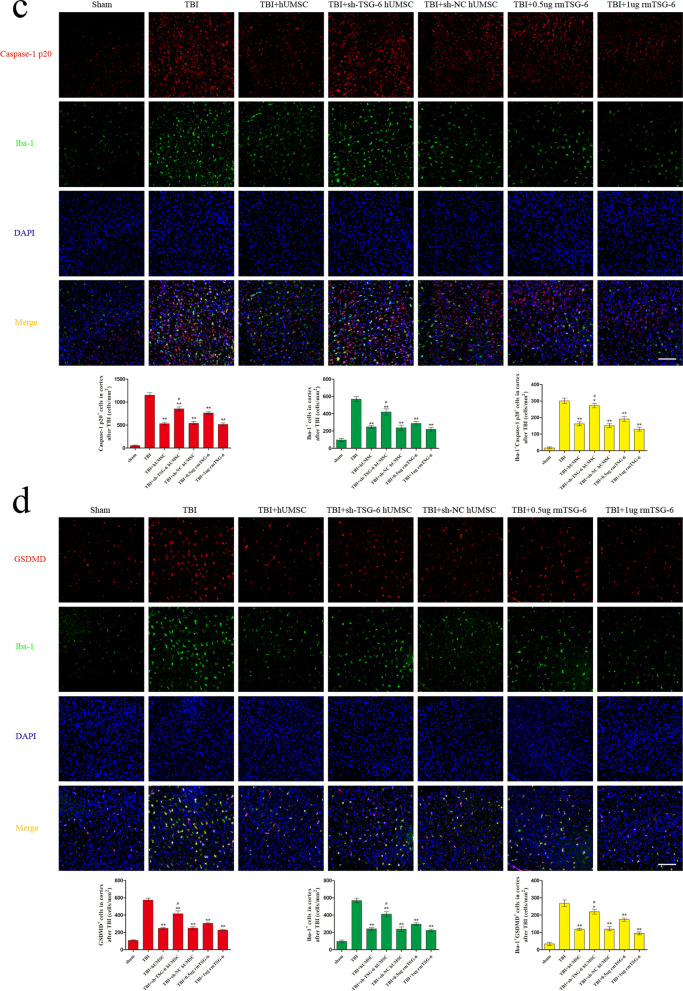
hUMSCs downregulated the NLRP3/Caspase-1/GSDMD signaling pathway and reduced microglia pyroptosis in TBI mice via TSG-6. TBI mice were intracerebroventricularly injected with 10 μL of PBS, 0.5 µg rmTSG-6 or 1 µg rmTSG-6, or 2.5 × 10^5^ hUMSCs, sh-TSG-6 hUMSCs, sh-NC hUMSCs, in 10 μL of PBS at 4 h after TBI surgery. Then TBI mice were sacrificed and brain tissues were removed at 48 h post-TBI surgery. **a** Representative protein bands and corresponding grayscale values of NLRP3, pro-Caspase-1, Caspase-1 p20, GSDMD, and cleaved-GSDMD performed by western blotting. **b**–**d** Representative double immunofluorescence staining photographs and quantification of Iba-1 (green) with NLRP3 (red), Caspase-1 p20 (red), or GSDMD (red) in the injured cortex. Scale bar = 50 μm. All data are represented as means ± SD (n = 6 mice/group) and compared by one-way ANOVA, followed by the *Tukey’s *post hoc test. *p < 0.05, **p < 0.01, versus TBI group; ^#^*p* < 0.05, sh-TSG-6 hUMSC group versus sh-NC hUMSC group

To assess whether TSG-6 secreted by hUMSCs exerts its anti-pyroptosis effect by inhibiting the NLRP3/Caspase-1/GSDMD signaling pathway in the brain cortex, we studied the levels of NLRP3, pro-Caspase-1, Caspase-1 p20, GSDMD, and cleaved-GSDMD by western blotting (Fig. 4a). As expected, compared with those in the sham group, the protein levels of NLRP3, Caspase-1 p20, GSDMD, and cleaved-GSDMD in the brain cortex were increased in TBI mice. In contrast, the TBI-induced upregulation of NLRP3, Caspase-1 p20, and cleaved-GSDMD was reduced following hUMSC transplantation or rmTSG-6 treatment. And the inhibitory effect of rmTSG-6 treatments on the expression of these proteins in the brain cortex of TBI mice was dose-dependent. However, TSG-6-shRNA transfection abrogated the inhibitory effect of hUMSCs on the expression of these proteins in the brain cortex. Whilst the expression of these proteins of TBI mice brains in the sh-NC hUMSC group and sh-TSG-6 hUMSC group were significantly different from each other, the sh-TSG-6 hUMSC group was still significantly different from the TBI group. To better understand the anti-pyroptosis effect of hUMSCs and TSG-6, we performed double immunofluorescence staining for Iba-1 and three important components of the pyroptosis signaling pathway: NLRP3, Caspase-1 p20, and GSDMD. As represented in Fig. 4b–d, the number of Iba-1+, NLRP3^+^, Caspase-1 p20^+^, and GSDMD^+^ cells in the brain cortex of TBI mice were significantly reduced following hUMSC transplantation or rmTSG-6 treatment. And the reduction effect of rmTSG-6 treatments on the number of Iba-1+, NLRP3+, Caspase-1 p20+, and GSDMD+ cells in the brain cortex of TBI mice was dose-dependent. However, transplantation with TSG-6 knockdown hUMSCs significantly increased the number of positive cells of Iba-1, NLRP3, Caspase-1 p20, and GSDMD as compared to that of the sh-NC hUMSC group. Whilst the number of Iba-1+, NLRP3+, Caspase-1 p20+, and GSDMD+ cells in the brain cortex of TBI mice brains in the sh-NC hUMSC group and sh-TSG-6 hUMSC group were significantly different from each other, the sh-TSG-6 hUMSC group was still significantly different from the TBI group. In addition, we found that hUMSC transplantation or rmTSG-6 treatment significantly reduced the number of NLRP3^+^ Iba-1^+^, Caspase-1 p20^+^ Iba-1^+^, and GSDMD^+^ Iba-1^+^ cells in the brain cortices of TBI mice, demonstrating the anti-pyroptosis effect of hUMSCs or TSG-6 in microglial cells. However, the anti-pyroptosis effect of hUMSCs was compromised when the expression of TSG-6 was inhibited by TSG-6-shRNA.

### hUMSCs inhibited pyroptosis in BV2 cells by secreting TSG-6

**Fig. 5:**
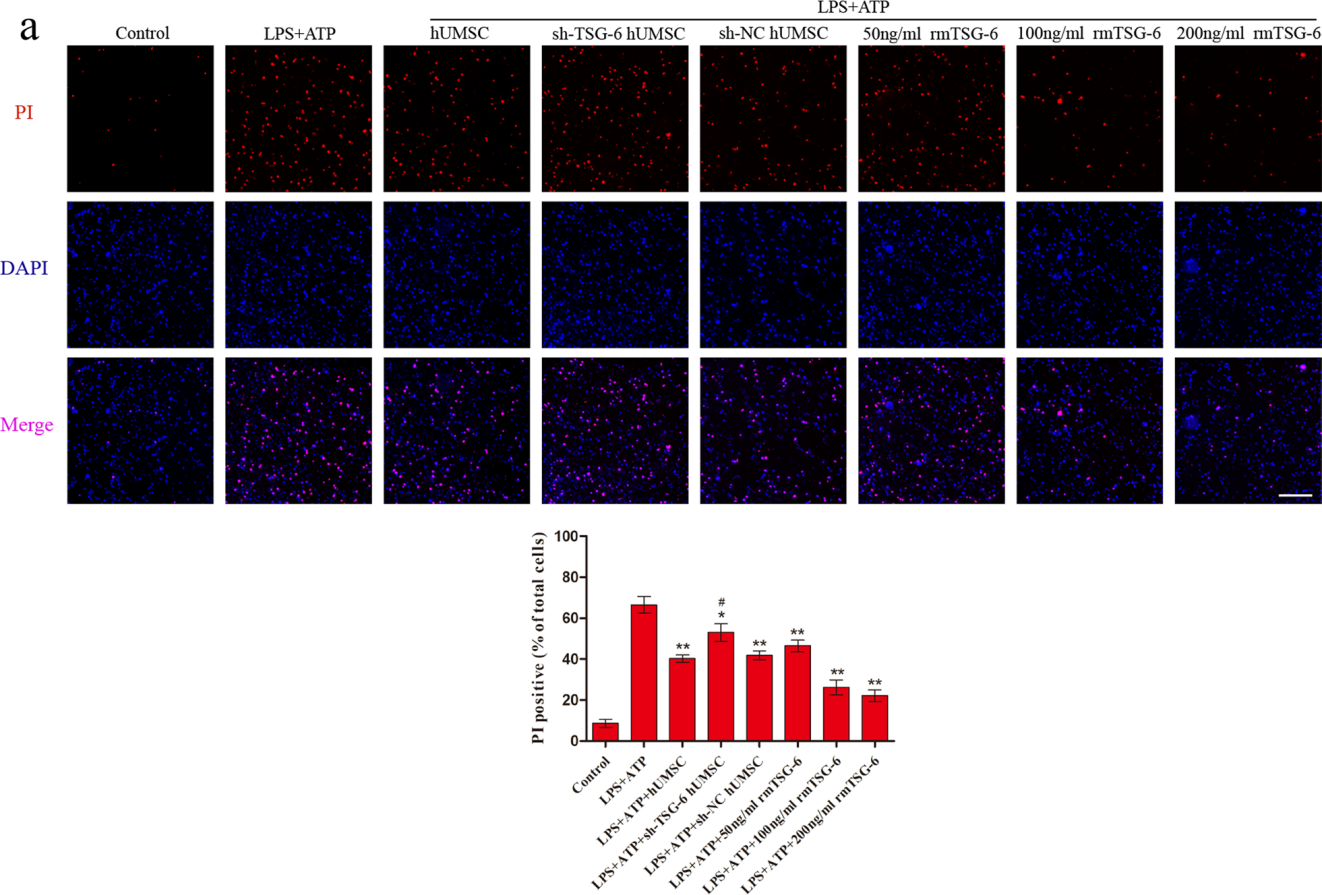

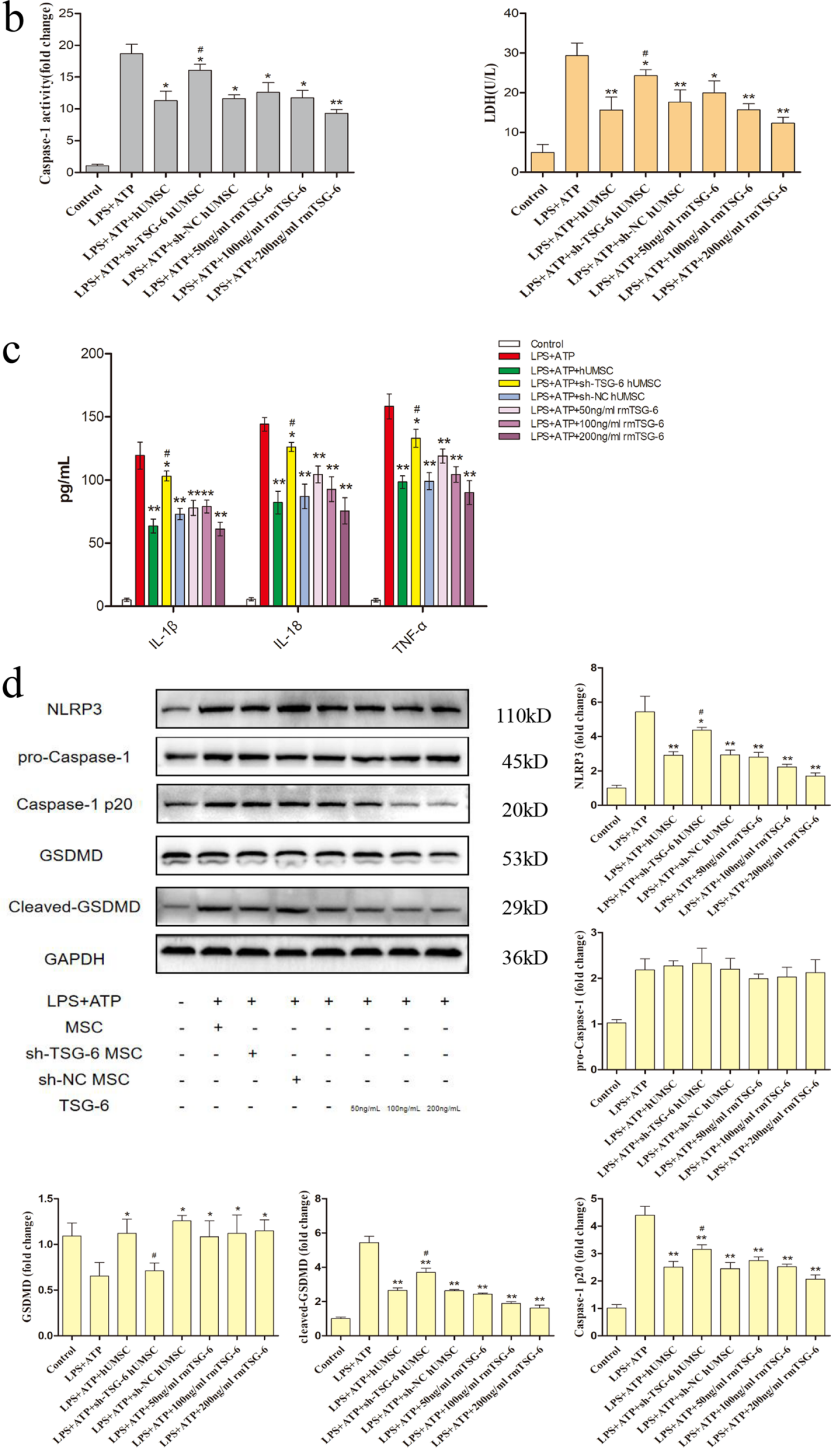
hUMSCs reduced LPS + ATP-induced BV2 microglia pyroptosis by secreting TSG-6. BV2 cells were co-cultured with hUMSCs, sh-TSG-6 hUMSCs, or sh-NC hUMSCs activated by TNF-α and different concentration of rmTSG-6 (50 ng/mL, 100 ng/mL, 200 ng/mL) and treated with or without LPS (1 µg/mL) + ATP (5 mM) for 12 h. **a** Representative photomicrographs and quantification of double staining with PI (red) and DAPI (blue) were captured by confocal microscopy. **b** Caspase-1 activity and the release of LDH in the supernatant was assessed using Caspase-1 activity and LDH activity detection kits. **c** The protein levels of IL-1β, pro IL-18, and TNF-α were evaluated by ELISA. **d** Representative protein bands and corresponding grayscale values of NLRP3, pro-Caspase-1, Caspase-1 p20, GSDMD and cleaved-GSDMD were performed by western blotting. Scale bar = 100 μm. All data are represented as means ± SD of at least 3 independent experiments and compared by one-way ANOVA, followed by the *Tukey’s *post hoc test. *p < 0.05, **p < 0.01, versus LPS + ATP group; ^#^*p* < 0.05, sh-TSG-6 hUMSC group versus sh-NC hUMSC group

We first assessed whether the expression of TSG-6 was increased in hUMSCs that had been stimulated by TNF-α (Additional file 4: Fig. S3). Thereafter, in order to gain a more comprehensive understanding of the effects of hUMSCs and TSG-6 on pyroptosis in microglial cells, LPS + ATP-induced BV2 microglia cells were co-cultured with different pretreated hUMSCs or different concentrations of rmTSG-6 for 12 h. After co-culture with normal hUMSCs or different concentrations of rmTSG-6, PI uptake levels (an indicator of cell death) of LPS + ATP-induced BV2 cells significantly decreased. And the reduction effect of rmTSG-6 treatments on PI uptake levels of BV2 cells was dose-dependent. However, after co-culture with TSG-6 knockdown hUMSCs, PI uptake levels of LPS + ATP-induced BV2 cells significantly increased as compared to those of the sh-NC hUMSC group. Whilst the PI uptake levels of BV2 cells in the sh-NC hUMSC group and sh-TSG-6 hUMSC group were significantly different from each other, the sh-TSG-6 hUMSC group was still significantly different from the TBI group (Fig. 5a). In addition, the data representing LDH release and Caspase-1 activity in the supernatant indicated that hUMSCs inhibited BV2 cell pyroptosis induced by LPS + ATP by the secretion of TSG-6 (Fig. 5b). Thereafter, we measured the protein levels of pro-inflammatory cytokines in the supernatant using an ELISA kit (Fig. 5c). Results demonstrated that BV2 cells co-cultured with hUMSCs or different concentrations of rmTSG-6 significantly decreased the protein levels of IL-1β, IL-18, and TNF-α in the supernatant. And the anti-inflammatory effect of rmTSG-6 treatments on BV2 cells was dose-dependent. However, the anti-inflammatory effect of hUMSCs was suppressed when the expression of TSG-6 was suppressed by TSG-6-shRNA. Whilst the protein levels of IL-1β, IL-18, and TNF-α in the supernatant of BV2 cells in the sh-NC hUMSC group and sh-TSG-6 hUMSC group were significantly different from each other, the sh-TSG-6 hUMSC group was still significantly different from the TBI group. Finally, to further assess whether TSG-6 secreted by hUMSCs exerts its anti-pyroptosis effect by inhibiting the NLRP3/Caspase-1/GSDMD signaling pathway in BV2 cells, we assessed the levels of NLRP3, pro-Caspase-1, Caspase-1 p20, GSDMD, and cleaved-GSDMD by western blotting (Fig. 5d). As expected, LPS + ATP-induced BV2 cells co-cultured with normal hUMSCs or different concentrations of rmTSG-6, protein levels of NLRP3, Caspase-1 p20, and cleaved GSDMD decreased significantly, but protein levels of GSDMD increased significantly. And the effect of rmTSG-6 treatments on these protein levels was dose-dependent. However, in BV2 cells co-cultured with TSG-6 knockdown hUMSCs, protein levels of NLRP3, Caspase-1 p20, and cleaved GSDMD increased significantly, but protein levels of GSDMD decreased significantly, as compared to those of the sh-NC hUMSC group. Whilst these protein levels of BV2 cells in the sh-NC hUMSC group and sh-TSG-6 hUMSC group were significantly different from each other, the sh-TSG-6 hUMSC group was still significantly different from the LPS + ATP group. Besides, the differences in protein levels of pro-Caspase-1 in LPS + ATP-induced BV2 cells between different treatments groups were not statistical significant.

## Discussion

TBI is a complicated pathological condition with unclear mechanisms. Studies have indicated that inflammation contributes to the development of TBI [45]. Pyroptosis, a novel mode of inflammation-associated cell death, has been implicated in several CNS pathologies, including TBI [16]. Pyroptosis-induced programmed cell death is distinguished from apoptosis in that it is mediated by the inflammation-associated protein Caspase-1 rather than the classical apoptosis-associated and Caspase-3 proteins. As a result, pyroptosis is also known as Caspase-1-dependent cell death [46]. In recent years, studies have suggested that MSCs may have protective effects in various disease contexts by regulating cell pyroptosis [29–32]. However, the effect of hUMSCs on TBI-induced pyroptosis remains to be determined. To assess whether hUMSCs inhibit pyroptosis and repair brain injury by secreting TSG-6, we obtained relevant experimental data following intracerebroventricular injection of hUMSCs with or without shRNA-mediated TSG-6 knockdown into a TBI mouse model. Results demonstrated that hUMSC transplantation or rmTSG-6 treatment significantly improved the neuromotor function of TBI mice, relieved cerebral edema, and decreased the number of TUNEL-positive cells in the brain, activity of Caspase-1 in the cerebral cortex, release of LDH in serum, and IL-1β, IL-18, and TNF-α levels in the injured cerebral cortex. The effect of hUMSCs with TSG-6 knockout on the above indices was weakened. Whilst the treatment effect in the sh-NC hUMSC group and sh-TSG-6 hUMSC group were significantly different from each other, the sh-TSG-6 hUMSC group was still significantly different from the TBI group. This may because of the incomplete knockdown of TSG-6 expression by the shRNA (Additional file 3: Fig. S2), and also indicate that other hUMSC-derived factors may also play a role. To further confirm the key role of TSG-6 in modulating above indices. TBI mice were intracerebroventricularly injected with different concentrations of rmTSG-6. TSG-6 supplementation largely reproduced the effects of the hUMSCs in a dose-dependent manner. These results demonstrate that hUMSC transplantation may inhibit cell pyroptosis and repair brain injury through TSG-6.

Next, We found that hUMSC transplantation or rmTSG-6 significantly inhibited the activation of the pyroptosis signaling pathway NLRP3/Caspase-1/GSDMD in the brain of TBI mice, but the inhibitory effect of hUMSCs was weakened by TSG-6-shRNA transfection. Increasing evidence has revealed that pyroptosis, as an important part of neuroinflammation, is triggered by inflammasome activation [47]. Stimulation of the cytoplasmic inflammasome complex (including NLRP3) activates Caspase-1 and translocates the gasdermin (GSDMD)-N domain to the cell membrane, resulting in pore formation and pyroptosis [48]. Previous study have demonstrated that NLRP3 inflammasome activation, ASC and caspase-1 expression, and IL-1β and IL-18 release were detected in the cerebral cortex of TBI rats [49]. Another study identified that the level of NLRP3 in the cerebrospinal fluid of patients with severe TBI was increased, suggesting that NLRP3 can be used as a prognostic indicator of TBI [50]. Therefore, the treatment of NLRP3 inflammasome-mediated post-TBI neuroinflammation and pyroptosis has garnered significant attention. It has been reported that propofol, mangiferin, and resveratrol can effectively inhibit NLRP3 inflammasome activation in a TBI rat model [51–53]. And studies have found that telmisartan and hyperbaric oxygen can also inhibit the activation of NLRP3 inflammasome in TBI mouse models [54, 55]. In addition, studies have also found that the NLRP3 inhibitors MCC950 and BAY11-7082 can reduce brain injury and inflammatory response, improve cognitive function in TBI mice, and result in a significant neuroprotective effect [56, 57]. These studies suggest that inhibiting NLRP3 inflammasome expression in the pyroptosis signaling pathway represents an effective treatment strategy for TBI. It has been reported that Caspase-1 deficiency or its inhibitors may prevent the inflammatory response of stroke, encephalomyelitis, TBI, and other neurological diseases and promote the repair of neurological function by reducing the expression of inflammasome and pyroptosis-related proteins IL-1β, IL-18, and GSDMD in the CNS [58–61]. The cleavage of GSDMD after combining with Caspase-1 is a key step in pyroptosis [15]. It has been shown that directly inhibiting or blocking the cleavage of GSDMD can significantly inhibit cell pyroptosis and improve neurobehavioral function, which can help preserve brain function [62–64]. As shown in Fig. 4, the TBI-induced upregulation of cleaved-GSDMD was reduced following hUMSC transplantation or rmTSG-6 treatment. And the inhibitory effect of rmTSG-6 treatments on the expression of cleaved-GSDMD in the brain cortex of TBI mice was dose-dependent. However, TSG-6-shRNA transfection abrogated the inhibitory effect of hUMSCs on the expression of cleaved-GSDMD in the brain cortex. These results demonstrate that hUMSC transplantation may inhibit GSDMD cleavage through TSG-6.

It has been reported that reduced activation of microglia can thus reduce inflammation and improve histological and functional outcomes in TBI rats [65]. Our previous study also reported that MSCs transplantation can effectively reduce microglial activation in TBI rat brain [41]. In this study, we found that hUMSC transplantation or rmTSG-6 treatment could significantly inhibit microglial activation in the brains of TBI mice. And the inhibitory effect of rmTSG-6 treatments on microglial activation in the brain cortex of TBI mice was dose-dependent. But hUMSCs transfected with TSG-6 knockdown shRNA significantly weakened the inhibitory effect of microglial activation. These results suggest that hUMSCs inhibit microglial activation through TSG-6 to protect the TBI mice brain. More importantly, studies have demonstrated that inhibition of pyroptosis in microglia improves CNS injury and contributes to neurological recovery [31, 66–68]. In a penetrating ballistic brain injury (PBBI) rat model, inflammasome activation and pyroptosis mainly occurred in microglia, suggesting that they may underpin the continuous pro-inflammatory state after PBBI. This result also suggests therapeutic targets for brain injuries [69]. In this study, Iba-1, a microglial marker, and pyroptosis signaling pathway proteins NLRP3/Caspase-1 p20/GSDMD were co-localized by double immunofluorescence staining, which revealed that increased expression of GSDMD coincided with Iba-1 staining. This suggested that cell pyroptosis mainly occurred in microglia. In addition, we found that hUMSC transplantation or rmTSG-6 treatment could significantly inhibit the pyroptosis of microglia in the brains of TBI mice, while hUMSCs transfected with TSG-6 knockdown shRNA significantly weakened the inhibitory effect of microglia pyroptosis. These results emphasize that hUMSCs inhibit microglial pyroptosis through TSG-6 and play a protective role in the brain.

Based on the aforementioned findings, we further probed the mechanisms by which hUMSCs inhibit microglial pyroptosis by secreting TSG-6. In our previous study, we revealed that MSCs inhibited the inflammatory response of BV2 microglia induced by lipopolysaccharide [42], enhanced M2 polarization of microglia, and alleviated neuroinflammation via TSG-6 [43]. These results suggest that TSG-6 secreted by MSCs is a powerful anti-neuroinflammatory factor. However, whether TSG-6 secreted by MSCs can regulate BV2 microglial pyroptosis remains unclear. This study clearly demonstrated that hUMSCs and rmTSG-6 could reduce the number of pyroptotic microglial cells and the expression of pyroptosis proinflammatory factors induced by LPS + ATP stimulation. More importantly, we found that both hUMSCs and rmTSG-6 inhibited the expression of active proteins in the LPS + ATP-induced pyroptosis signaling pathway NLRP3/Caspase-1/GSDMD, whereas hUMSCs with TSG-6 knockdown significantly weakened the regulation of pyroptosis in BV2 microglia. These results suggest that hUMSCs exert their anti-pyroptosis effects on BV2 microglial cells via TSG-6.

## Conclusion

hUMSCs inhibited pyroptosis, especially microglial pyroptosis, by dampening the activation of the NLRP3/Caspase-1/GSDMD signaling pathway through TSG-6. This represents one of the key mechanisms behind the effectiveness of hUMSC transplantation in TBI treatment. Furthermore, our results suggested that intracerebroventricular administration of exogenous recombinant TSG-6 may represent a viable therapy option for TBI-induced pyroptosis.

## Supplementary Information

**Additional file 1: Fig. S1.** Surface marker expression in hUMSCs. hUMSCs were confirmed by flow cytometry analysis following three passages as positive for CD73 (100%), CD105 (99.96%), and CD90 (100%), and negative for CD45 (0.06%), HLA-DR (0.06%), CD34 (0.01%), CD11b (0.01%), and CD19 (0.00%).

**Additional file 2: Fig. S2.** The expression of the TSG-6 was knocked down using TSG-6-shRNA lentivirus. The relative mRNA expression levels of TSG-6 mRNA were determined by RT-qPCR and representative protein bands and corresponding grayscale values of TSG-6 were determined by western blotting. All data are represented as means ± SD of at least 3 independent experiments and compared by one-way ANOVA, followed by the *Tukey’s *post hoc test. **p < 0.01 versus control or control shRNA.

**Additional file 3: Fig. S3.** hUMSCs overexpressed TSG-6 in response to the inflammatory cytokine TNF-α. The relative expression levels of TSG-6 mRNA were determined by RT-qPCR and representative protein bands and corresponding grayscale values of TSG-6 were performed by western blotting. All data are represented as means ± SD of at least 3 independent experiments and compared by *t*-test. **p < 0.01 versus control.

## Data Availability

Data and material are available upon request.

## Abbreviations

TBI: Traumatic brain injury
MSCs: Mesenchymal stem cells
hUMSCs: Human umbilical cord mesenchymal stem cells
TSG-6: Tumor necrosis factor α stimulated gene/protein 6
CNS: Central nervous system
LPS: Lipopolysaccharide
ATP: Adenosine triphosphate
NLRP3: Nod-like receptor protein 3
Caspase-1: Cysteinyl aspartate specific proteinase 1
GSDMD: Gasdermin D
IL: Interleukin
PBBI: Penetrating ballistic brain injury
