# Supplementary figures and images for: Mesenchymal stem cells protect against TBI-induced pyroptosis in vivo and in vitro through TSG-6
Source: Cell Commun Signal. 2022 Aug 18;20:125. doi: 10.1186/s12964-022-00931-2 (PMC9387023; doi:10.1186/s12964-022-00931-2)

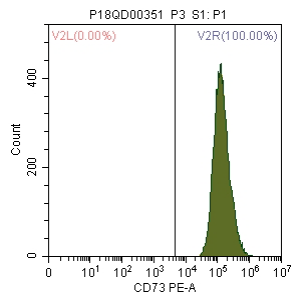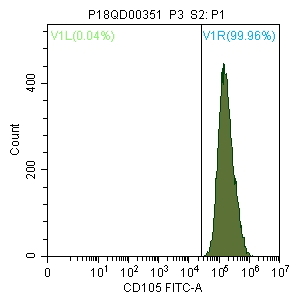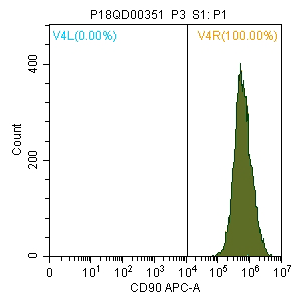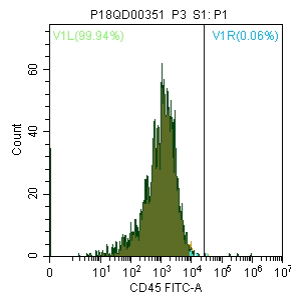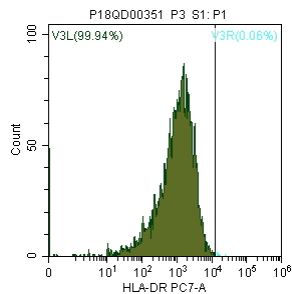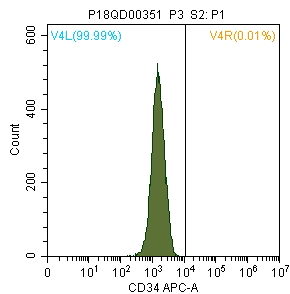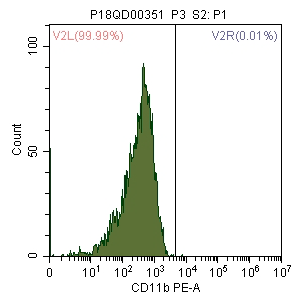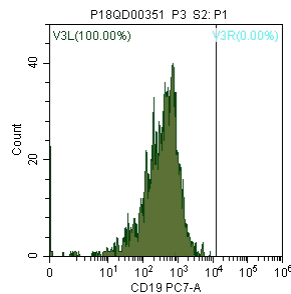

Supplement: Supplementary file 2 — Additional file 1: Fig. S1. Surface marker expression in hUMSCs. hUMSCs were confirmed by flow cytometry analysis following three passages as positive for CD73 (100%), CD105 (99.96%), and CD90 (100%), and negative for CD45 (0.06%), HLA-DR (0.06%), CD34 (0.01%), CD11b (0.01%), and CD19 (0.00%). [file 12964_2022_931_MOESM2_ESM.pdf]

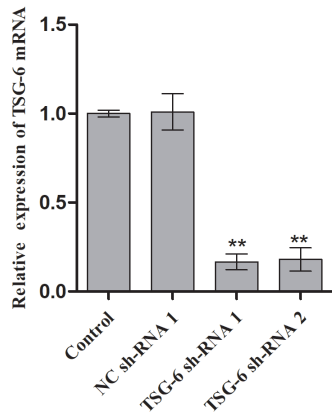

TSG-6

GAPDH

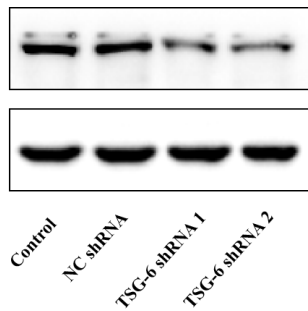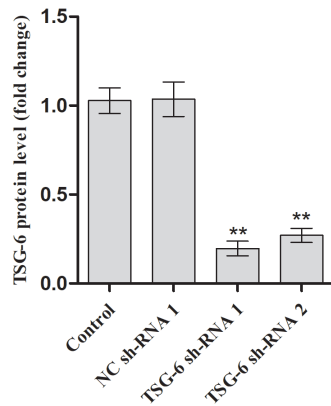

Supplement: Supplementary file 3 — Additional file 2: Fig. S2. The expression of the TSG-6 was knocked down using TSG-6-shRNA lentivirus. The relative mRNA expression levels of TSG-6 mRNA were determined by RT-qPCR and representative protein bands and corresponding grayscale values of TSG-6 were determined by western blotting. All data are represented as means ± SD of at least 3 independent experiments and compared by one-way ANOVA, followed by the Tukey’s post hoc test. **p < 0.01 versus control or control shRNA. [file 12964_2022_931_MOESM3_ESM.pdf]

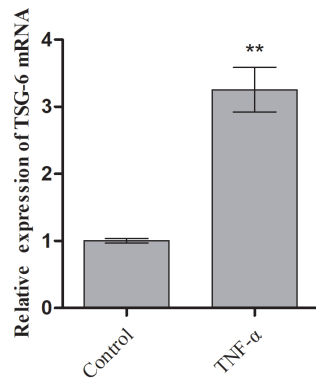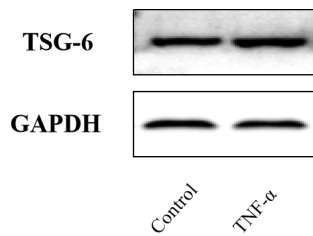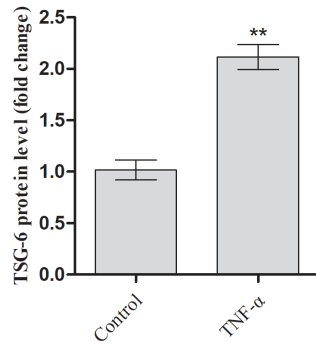

Supplement: Supplementary file 4 — Additional file 3: Fig. S3. hUMSCs overexpressed TSG-6 in response to the inflammatory cytokine TNF-α. The relative expression levels of TSG-6 mRNA were determined by RT-qPCR and representative protein bands and corresponding grayscale values of TSG-6 were performed by western blotting. All data are represented as means ± SD of at least 3 independent experiments and compared by t-test. **p < 0.01 versus control. [file 12964_2022_931_MOESM4_ESM.pdf]
